# Investigation of the optimum calcination temperature for water treatment plant sludge to develop a sustainable alkali activated concrete

**DOI:** 10.1038/s41598-025-85225-6

**Published:** 2025-01-16

**Authors:** Jasmin Osama Abdelhalim, Ismail Amer, Ibrahim Abdel Latif, Ihab Fawzy, Sayed Ismail, Mohamed A. Khalaf

**Affiliations:** 1 Structural Engineering Department, Faculty of Engineering, Ain Shams University, Cairo, Egypt; 2 Public Works Department, Faculty of Engineering, Ain Shams University, Cairo, Egypt

**Keywords:** Water treatment sludge, Calcination, Chapelle test, Compressive strength, Temperature, Alkali-activated concrete., Mechanical properties, Civil engineering

## Abstract

Nowadays, Egypt is treating the Nile River Water to produce drinking water, and this process generates large amounts of waste, around 635 million m^3^ annually, which is called water treatment plant sludge (WTPS). This WTPS cost the government around 30 million US dollars to return it back to the Nile River in addition to negatively affecting the environment. Therefore, there is an urgent need to find environmentally friendly alternatives that reduce the impact of such an issue. This paper focuses on treating WTPS by drying, grinding and calcining to develop it as an alternative binder for use in alkali-activated concrete. This approach would not only provide green construction material but also reveal an environmental disposal route for the sludge produced in Egypt or in any country has the same issue. The treatment methodology used in this study was based on finding the optimum calcination temperature regime for WTPS after drying and grinding. Fifteen specimens of WTPS powder were used to investigate the optimum calcination temperature and duration by applying different temperatures ranging from 500 °C to 800 °C for various exposure durations of 30, 60 and 90 min. XRD and Chapelle tests were employed to chemically investigate the efficiency of the obtained calcined WTPS specimens, while strength activity index and compressive strength tests were used to mechanically verify the findings of the chemical tests. The results indicated that the calcination regime, which involved applying a maximum temperature of 650 °C for 90 min, achieved the best chemical characteristics and a strength activity index of 145%. Moreover, this regime resulted in a compressive strength of 21 MPa when WTPS powder was used as a precursor in alkali-activated concrete. Additionally, this paper presented a brief comparison of the production cost and energy consumption between cement and WTPS. The comparison demonstrated the efficiency of using WTPS as a replacement for cement, showing that the production of WTPS costs 50% less and consumes 92% less energy than cement.

## Introduction

Cement is considered the most commonly used material for construction applications due to its raw material availability and its superior mechanical properties. The production of cement contributes approximately 8% of the total carbon dioxide emissions^1^. Additionally, cement production can cause several other environmental issues, such as high energy usage for the calcination of limestone^2^. Therefore, it is highly demanding to find new innovative green building materials that mitigate or alleviate environmental problems^3,4^. Alkali-activated materials composed of an aluminosilicate source and an alkaline activator are now being investigated as promising alternatives to cement in concrete manufacturing. The aluminosilicate source can be a raw material or an industrial residue of low added value in a powdered form, and when mixed with alkali activators such as carbonates, silicates, alkaline sulfates, or a solution of hydroxides, it can provide superior mechanical properties^4,5^. One of the sources of aluminosilicates is Water treatment plant sludge (WTPS) which is produced from the coagulation and flocculation processes of Nile River water treatment^6^. Annually, 635 million m^3^ of WTPS is produced in Egypt, and this quantity is estimated by the (CAPMAS, 2022) to double by the year 2078 to meet the growing demand of the population for safe drinking water. WTPS is considered a waste, and countries try to find different ways for its disposal. For example, the WTPS in the Netherlands is disposed of in landfills, which have operational costs between 30 and 40 million U.S. dollars per year^6^. In Egypt, the WTPS is returned to the Nile River, costing the government approximately 30 million U.S. dollars annually and consequentially increasing the future treatment process cost. So, treating WTPS, which is composed mainly of silica, aluminum and iron hydroxide makes it a good material for producing alkali-activated materials and provides a safe and environmentally friendly disposable route for WTPS^6^. Also, this confirms more than one of the sustainable development goals (SDGs)^7^.

The WTPS has been used as a replacement for cement or clay in the production of building bricks^8–12^. Hegazy et al. (2012) concluded that mixing 75% of WTPS with 25% rice husk and firing at 1200 °C gave an optimum mix for the replacement of clay for the production of bricks^8^. Additionally, Tantawy et al. (2017) reported that replacing 15–30% of clay with WTPS would give a brick with a compressive strength close to the strength of a brick that was produced from clay alone^9^. Ibrahim et al. (2021) replaced kaolin with WTPS for the production of bricks and reported that increasing the WTPS by more than 60% caused dimension changes^11^.

Additionally, WTPS was used for the production of cement mortar, concrete and fine aggregates^13–18^. Haider et al. (2013) concluded that replacing 6% of cement with WTPS enhanced the compressive strength of concrete^15^. Didamony et al. (2014) reported that 5% of WTPS and slag were optimal for the replacement of cement^16^.

In 2017, Geraldo et al. mixed WTPS with sodium silicate solution to replace 0, 15, 30 and 60% of the metakaolin material to produce geopolymers. The research recorded a compressive strength of 28 MPa at 28 days for the 15% replacement mixture^17^. Hagemann et al. (2019) used WTPS with limestone to replace Portland cement. The authors treated WTPS-like clay as a constituent of silica, aluminum, and iron with the aim of obtaining the same mechanical properties as those of clay. Activated WTPS was determined to be the optimum treatment by drying, calcining at 700 °C for 1 h and grinding for 1 h. The researchers used the Chapelle test and strength activity index test to confirm their findings. The study concluded that the optimum mixture is 15% replacement of treated WTPS for cement, for which the 28 day compressive strength is 80 MPa^18^.

The use of the WTPS in construction applications showed superior properties to those of clay or cement. However, the use of the WTPS has been limited due to concerns related to the variability of the physical properties of the WTPS products, which was due to the variability in the chemical composition and water and organic matter contents of the WTPS. Additionally, researchers have found that to produce bricks with high amounts of WTPS, high sintering temperatures are needed, leading to a more energy-intensive process^6,19^.

However, very few studies have evaluated the activation of WTPS through calcination or mixing with an activator solution with the aim of producing green alkali activated material. Therefore, the activation of WTPS (aluminosilicate source) through grinding and firing is considered an environmentally friendly solution for construction applications because it provides a safe disposable route for WTPS in addition to providing a green binder material.

This paper aimed to determine the optimum treatment calcination regime in terms of temperature and exposure duration for WTPS to be used as a precursor in alkali-activated concrete by measuring the pozzolanic reactivity of the treated WTPS powder. Additionally, the material phase changes that took place at different calcination temperature and duration were assessed. Then, the mechanical characteristics of the treated WTPS, in terms of strength activity index and compressive strength, were assessed. In addition, an economic and energy feasibility study was conducted to verify the adequacy of replacing cement with WTPS in terms of both cost and energy consumption.

## Experimental program

### Materials

In this study, ordinary Portland cement (OPC), ground granulated blast furnace slag (GGBFS) and WTPS were utilized as binders. The OPC used was grade 42.5 N in accordance with BS EN 197-1^20^. The specific gravity of the GGBFS used was 2.8. The chemical compositions of the OPC and GGBFS used are listed in Table 1. A locally available WTPS was used. The WTPS was obtained from the 6th of October Water Treatment Plant, Giza, Egypt. The delivered raw WTPS was first dewatered by drying in an oven at 90 °C for 24 h. Then, the samples were ground in ball mills to increase the fineness of the WTPS. The recorded fineness of the prepared WTPS powder, expressed in terms of specific surface area, was 6750 cm^2^/gm according to the ASTM C204-07 according to the Blaine test^21^. The specific gravity of the prepared sludge powder was measured according to ASTM C188-17^22^, and it was 2.64.

**Fig. 1:**
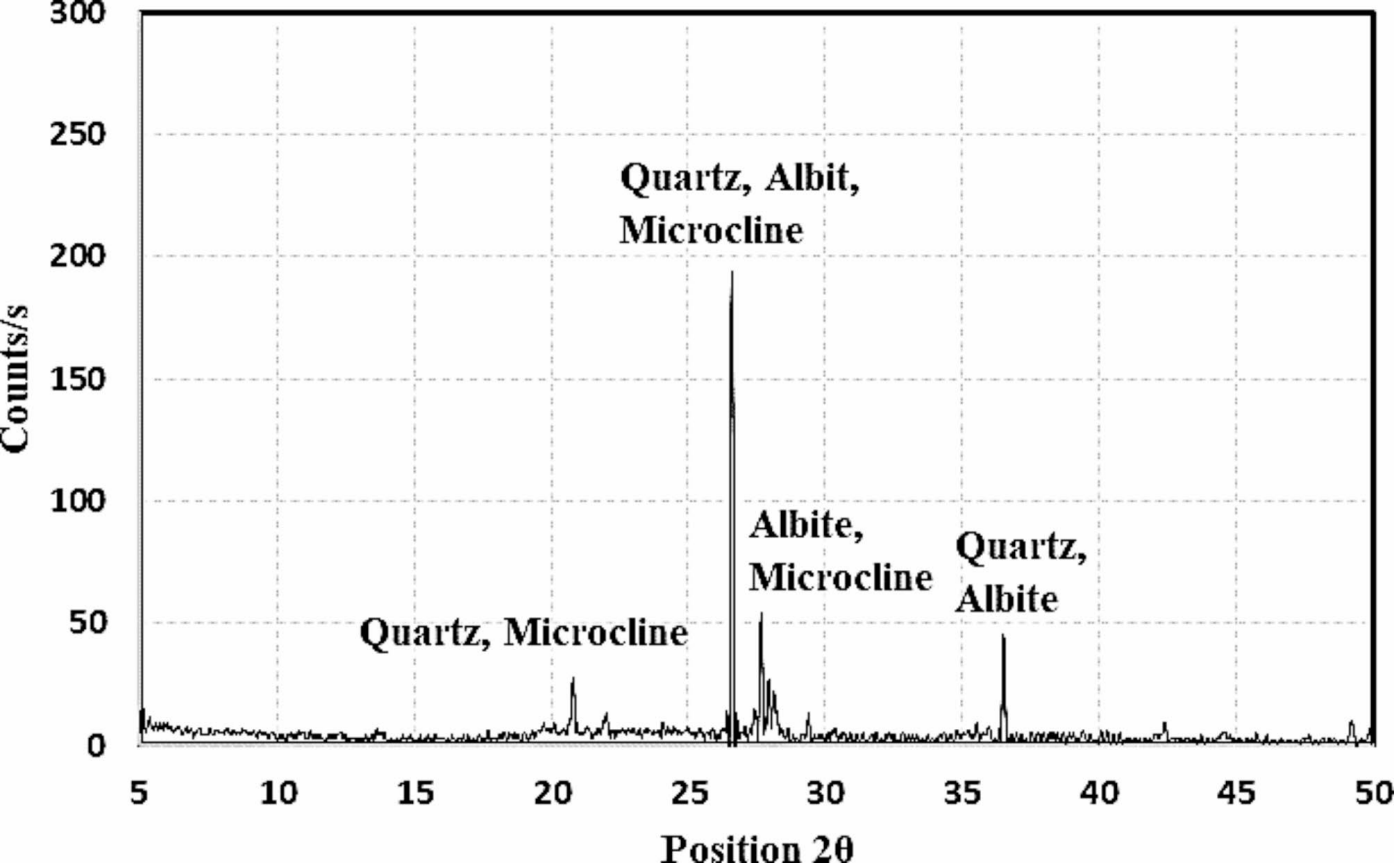
XRD results of the in-nature WTPS powder (before calcination).

**Fig. 2:**
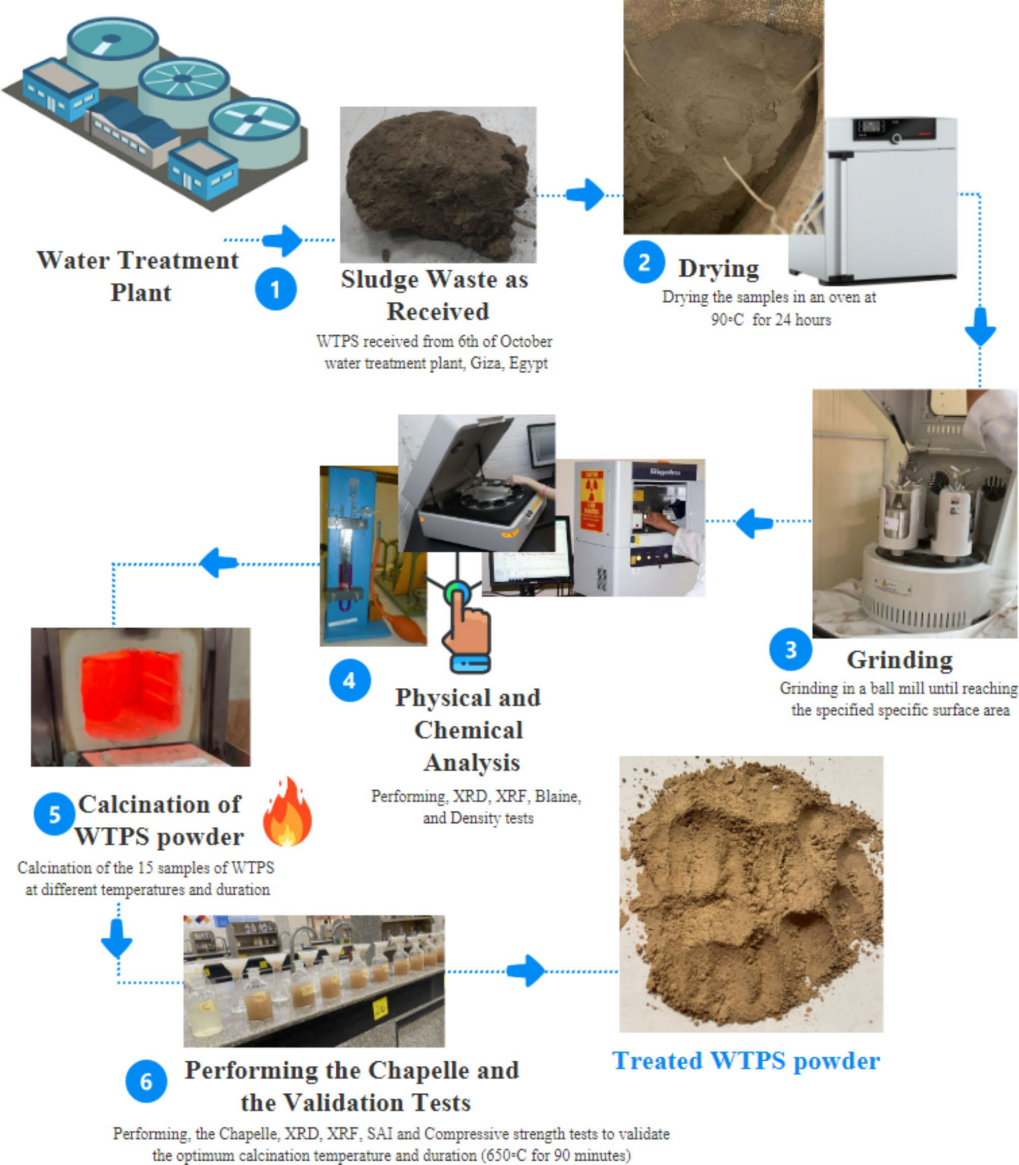
Schematic of the full preparation cycle of the WTPS powder.

Afterwards, XRD and XRF tests were also performed to assess the chemical composition of the prepared WTPS powder. X-ray fluorescence (XRF) was performed using an Xios stylePW-1400. The XRF results are presented in Table 2. In the XRF test, 10 g of the WTPS specimen was dried in an oven for 24 h to remove moisture and then mixed with 1 g of borax (filling material). Finally, the mixture was placed in a lead ring and compressed at 3–12 ton/cm^2^ to be shaped into a tablet to be ready for analysis. The results showed that the two components with the highest values were SiO_2_ (52.00%) and Al_2_O_3_ (14.30%). with a total percentage of approximately 66.3%, which was a good indicator that the WTPS powder was a precursor in the alkali-activated materials. X-ray diffraction (XRD) was performed using an X׳Pert PRO PAN analytical X-ray diffraction equipment model with a secondary monochromator and Cu radiation (λ = 1.542 Å) at 45 K.V. and 35 M.A. and a scanning speed of 0.04 ^°^/sec. The diffraction peaks between 2θ = 5^°^ and 50° were obtained. The results of the XRD test, which are presented in Fig. 1, show that the WTPS powder is crystalline, with peaks resembling the presence of quartz (SiO_2_), microcline (K Al Si_3_ O_8_), and albite (Na_0.98_ Ca_0.02_ (Al_2_ Si_2_O_5_ O_8_) at 2θ values equal to 20.87, 26.61, 27.71 and 36.47, respectively. The highest peak is at 2θ = 27.71° with 194 counts. The full preparation cycle for the WTPS powder is illustrated schematically in Fig. 2. The fine aggregate used was natural sand with a fineness modulus of 2.70. The activator used in this study was a mixture of sodium silicate (SS) and sodium hydroxide (SH). SH was in flake form, while SS was in liquid form, and both were obtained from a local producer. The activator solution was prepared by dissolving the SH flakes in potable water and then adding the SS to the solution. The activator solution was left to cool until it reached room temperature before it was used in the mixtures. The chemical compositions by mass of the SH and SS used were 60.25% Na_2_O and 39.75% H_2_O and 31.0% SiO_2_, 12.0% Na_2_O and 57.0% H_2_O, respectively.

**Table 1:** Chemical composition of the OPC and GGBFS (mass %).

Component	SiO_2_	Al_2_O_3_	Fe_2_O_3_	CaO	MgO	K_2_O	Na_2_O	SO_3_	TiO_2_	Mn_2_O_3_
OPC	20.13	5.32	3.61	61.63	2.39	0.13	0.37	2.87	–	–
GGBFS	41.66	13.96	1.49	34.53	5.53	0.97	0.49	–	0.58	0.35

**Table 2:** XRF results of the in-nature WTPS powder (before calcination).

Components	SiO_2_	Al_2_O_3_	Fe_2_O_3_	CaO	MgO	Na_2_O	K_2_O	SO_3_	TiO_2_	*P*_2_O_5_	MnO	LOI
Mass %	52.00	14.30	7.42	4.39	0.97	0.25	0.80	0.86	1.23	0.50	0.36	16.30

### WTPS calcination methodology

**Fig. 3:**
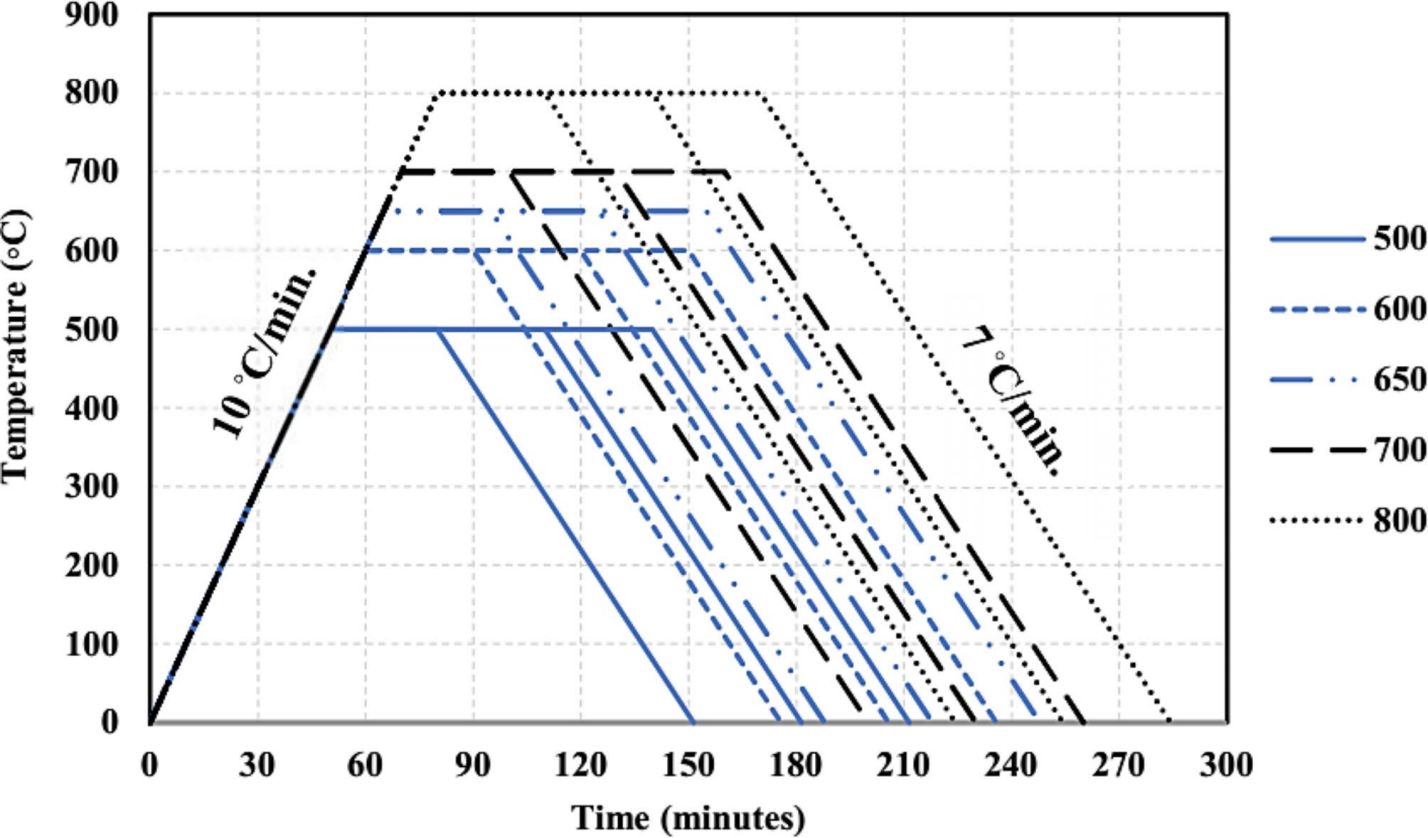
Heating regime applied to the WTPS specimens.

The calcination methodology applied in this study was based on determining the optimum temperature and exposure duration that provide the best chemical and mechanical characteristics for the use of WTPS powder as a precursor for alkali-activated concrete. Therefore, five different elevated temperatures of 500, 600, 650, 700 and 800 °C were tested with three different exposure durations of 30, 60 and 90 min for each temperature. The selected temperatures and durations were chosen according to Hagemann et al. (2019) previous research on calcined WTPS. Based on the selected temperatures and durations, 15 WTPS specimens were calcined, as shown in Table 3. The prepared WTPS specimens were exposed to the specified elevated temperatures in an electric furnace, as shown in Fig. 2, with a temperature capacity of 1200℃. The heating rate of the furnace was 10 °C/min. After reaching the required temperature, the heated specimen was kept at this temperature in the furnace for the specified period. Then, the heated specimen was cooled to ambient temperature. Figure 3 shows the heating regimes at all the specified elevated temperatures for the different application durations.

**Table 3:** Description of the calcined WTPS specimens.

Ser.	Specimen Code	Calcination temperature (^℃^)	Exposure duration (min.)
1	500−30	500	30
2	500−60	500	60
3	500−90	500	90
4	600−30	600	30
5	600−60	600	60
6	600−90	600	90
7	650−30	650	30
8	650−60	650	60
9	650−90	650	90
10	700−30	700	30
11	700−60	700	60
12	700−90	700	90
13	800−30	800	30
14	800−60	800	60
15	800−90	800	90

### Test method

To investigate the efficiency of the obtained calcined WTPS specimens and to determine the optimum calcination methodology, all the calcined WTPS specimens were tested chemically by the Chapelle method, by XRD and XRF tests and mechanically by the strength activity index and compressive strength tests.

#### Chapelle test

**Fig. 4:**
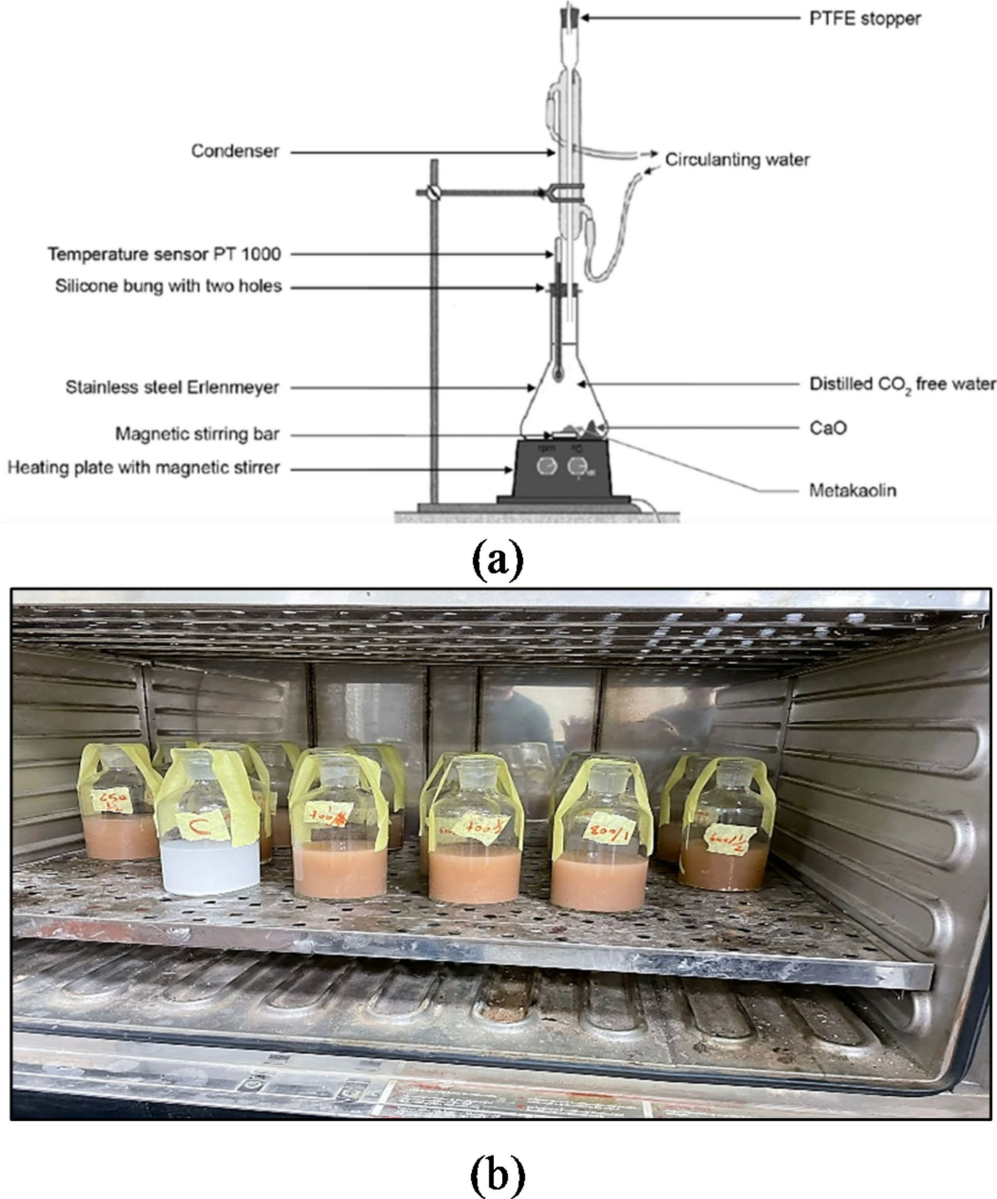
Chapelle test. (**a**) Standard apparatus^24^. (**b**) Test specimens in the oven for drying.

The Chapelle test was used to assess the pozzolanic reactivity of the tested WTPS specimens according to NF P 18–513 (2010)^23^. This test assesses pozzolanic activity through the determination of the amount of lime consumed by pozzolanic reactions. A mixture of WTPS powder and CaO was mixed and kept at 90 °C in a Chapelle test standard apparatus for 16 h. Then, the amount of CaO that did not react was measured. Figure 4a shows the standard apparatus used for the test^24^. Therefore, after dewatering and grinding, each of the 15 WTPS powder specimens was calcined at the required temperature for the required exposure duration, after which each specimen was quenched by sudden air cooling. Through this test, the amount of Portlandite (Ca(OH)_2_) was measured after it was consumed by 1 g of WTPS combined with 2 g of CaO and 250 ml of distilled water. This mixture was continuously stirred while the mixture was heated in an oven at 90 °C for 24 h. Figure 4b shows the test specimens prepared in the drying oven.

**Fig. 5:**
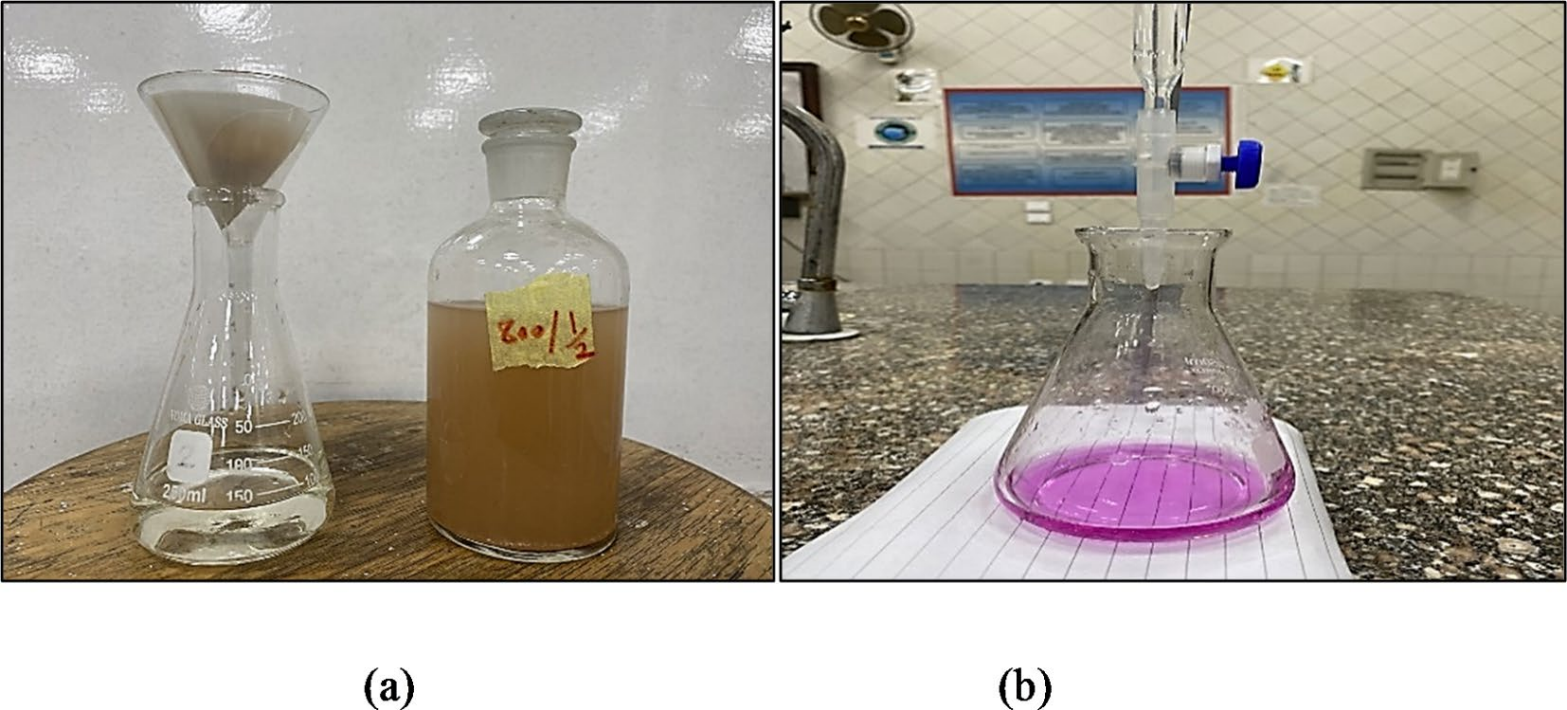
Filtration process. (**a**) Specimen (800−30). (**b**) Titration process using phenolphthalein indicator.

After removing the test specimens from the oven and allowing them to cool at ambient temperature, the amount of free Portlandite was quantified via sucrose extraction and acid titration. The sucrose solution was prepared by dissolving 60 g of sugar in 250 ml of distilled water. The sucrose solution was added to the prepared mixture and stirred using a magnetic stirrer for 15 min. The mixture was filtered, and 25 ml of the filtrate was titrated with 0.1 N hydrogen chloride (HCl) by using a phenolphthalein indicator. Figure 5 shows the filtration process and titration process using the phenolphthalein indicator. An average of three readings were taken for each WTPS specimen from the 15 prepared specimens to obtain an accurate volume of HCL, which was used in the titration process.

The pozzolanic activity of the tested WTPS specimens was calculated using the following.

formula^22^:

$$PAS\,=\,2 \times \left( {V1 - V2/V1} \right) \times \left( {74/56} \right) \times 1000$$

PAS is the pozzolanic activity of the tested WTPS specimens (mg CaO consumed/g WTPS); V1 is the volume of 0.1 N HCl (ml), which is necessary for the titration of 25 ml of the filtrated solution obtained without WTPS; V2 is the same but for the solution obtained with WTPS; and 74 and 56 are the molecular weights of Ca(OH)_2_ and CaO, respectively. The recorded average V1 and V2 for the 15 WTPS specimens are substituted in the above equation, and the resulting PAS value is compared to the value of 700. A PAS greater than 700 indicates that a pozzolanic reaction occurred. The highest PAS value recorded resembled the best pozzolanic reactivity, which was the optimum calcination temperature and exposure duration for WTPS.

#### XRD test

XRD was also conducted to assess the changes in the material phases of the WTPS powder specimens calcined at different temperatures and for different exposure durations. This test was used to verify the results obtained by the Chapelle test. Three specimens were selected based on the obtained PAS values; these three specimens had the highest, average and lowest PAS values.

#### XRF test

An XRF test was used to verify the findings of the Chapelle test chemically, where the chemical composition of the specimen, which recorded the highest PAS value, was examined and compared to both its XRD results and the XRF test obtained for the in-nature specimen.

#### Strength activity index test

The strength activity index test was carried out to assess the mechanical behavior of the calcined WTPS powder by testing specimens that contained 20% WTPS plus 80% cement as a binder during compression and comparing the obtained compressive strength with that of a control mixture (100% cement without WTPS). If the compressive strength of the specimens, which contain WTPS, reaches 75% or more of the compressive strength of the control mix, then the WTPS is considered an active pozzolan according to ASTM C 618^25^. Therefore, this test was used to verify that the WTPS specimen, which had the highest recorded PAS value, exhibited good mechanical behavior. The strength activity index test was carried out according to ASTM C311^26^, where two mortar mixes were prepared, a control mixture (100% cement) and a test mixture with 20% replacement of cement with the calcined WTPS powder, which recorded the highest PAS value. Table 4 presents the mix proportions for the tested mixes, which were designed according to ASTM C109^27^. These mixes were tested under compression after 7 days using cubic specimens with dimensions of 50 × 50 × 50 mm according to ASTM C109^27^.

**Table 4:** Mix proportions of the tested mixtures.

Mix code	Cement (g)	WTPS (g)	Sand (g)	Water (ml)
Control mix	500	–	1375	242
Test mix	400	100	1375	242

#### Compressive strength test

The compressive strength test was also used to investigate the mechanical behavior of the calcined WTPS powder, but this study used the WTPS powder as a precursor in alkali-activated mortars. The WTPS and GGBFS powders were used as precursors in the performed alkali-activated mortars, and the activator solution used was a combination of sodium silicate (SS) and sodium hydroxide (SH). All mixing parameters and their levels were specified based on the literature review^4,28–33^. The four mortar mixes were investigated with the parameters and levels that are presented in Table 5. The proportions of the four mortar mixes were designed according to ASTM C109^27^, as listed in Table 6. The four mixes were tested under compression at the ages of 3 days, 7 days and 28 days using cubic specimens with dimensions of 50 × 50 × 50 mm according to ASTM C109^27^.

**Table 5:** Parameters and levels of the mixtures used.

Mix code	WTPS: GGBFS	Na_2_O^a^ (%)	Ms^b^	W/B^c^	Curing conditions
Mix 1	100:0	12	1.9	0.5	Ambient temperature
Mix 2	100:0	12	1.0	0.5	Ambient temperature
Mix 3	100:0	12	1.9	0.5	Heat curing (90℃)–4 h
Mix 4	50:50	12	1.0	0.5	Ambient temperature

^a^Percentage relative to the binder content.

^b^Ms = SiO_2_/Na_2_O.

^c^W/B = Water-to-binder ratio.

**Table 6:** Mix proportions of the performed mortar mixes.

Mix code	WTPS (g)	GGBFS (g)	Sand (g)	SS (g)	SH (g)	Water (ml)
Mix 1	750	0	2062.5	551.6	39.5	44.9
Mix 2	750	0	2062.5	290.3	91.5	173.1
Mix 3	750	0	2062.5	551.6	39.5	44.9
Mix 4	375	375	2062.5	290.3	91.5	173.1

## Results and discussion

**Fig. 6:**
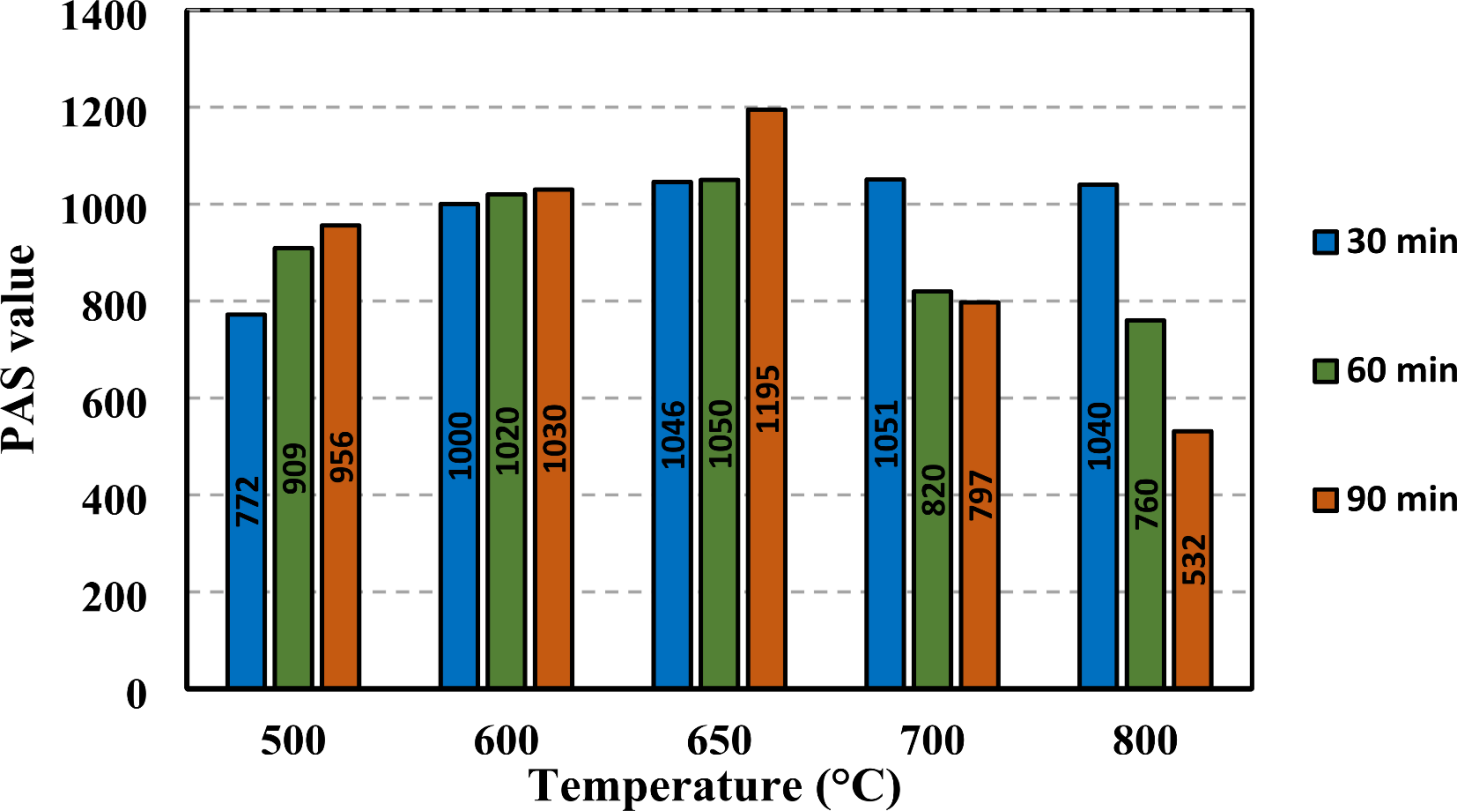
The obtained PAS values for all the calcined WTPS test specimens.

The pozzolanic activity (PAS) was calculated for all the WTPS specimens previously described in Table 3 and in addition to another WTPS specimen without calcination, which was used as a control specimen. Figure 6 shows the obtained PAS values for all the tested specimens. The figure shows that the PAS value for all the tested specimens exceeded 700, which means that there was good pozzolanic reactivity except for the tested specimen (800−90), where the PAS value was less than 700, indicating that there was no pozzolanic reactivity. This may be due to the recrystallization of the particles when they are exposed to high temperatures for a long duration^34^. For 500 and 600 °C, increasing the calcination exposure duration increased the pozzolanicity. Additionally, at 650 °C, the PAS increased with increasing exposure duration, reaching the highest PAS of 1194 recorded for test specimens (650−90). Above these temperatures, the PAS value decreased with increasing temperature and duration. Additionally, Fig. 6 shows the different trends in the exposure durations versus the different temperatures obtained with the recorded PAS values for each specimen. The 30 min exposure duration had an almost steady trend, while the 60 min and 90 min exposure durations had an increase in pozzolanic activity, reaching a peak at 650 °C and then a decreasing trend from the peak until 800 °C.

**Fig. 7:**
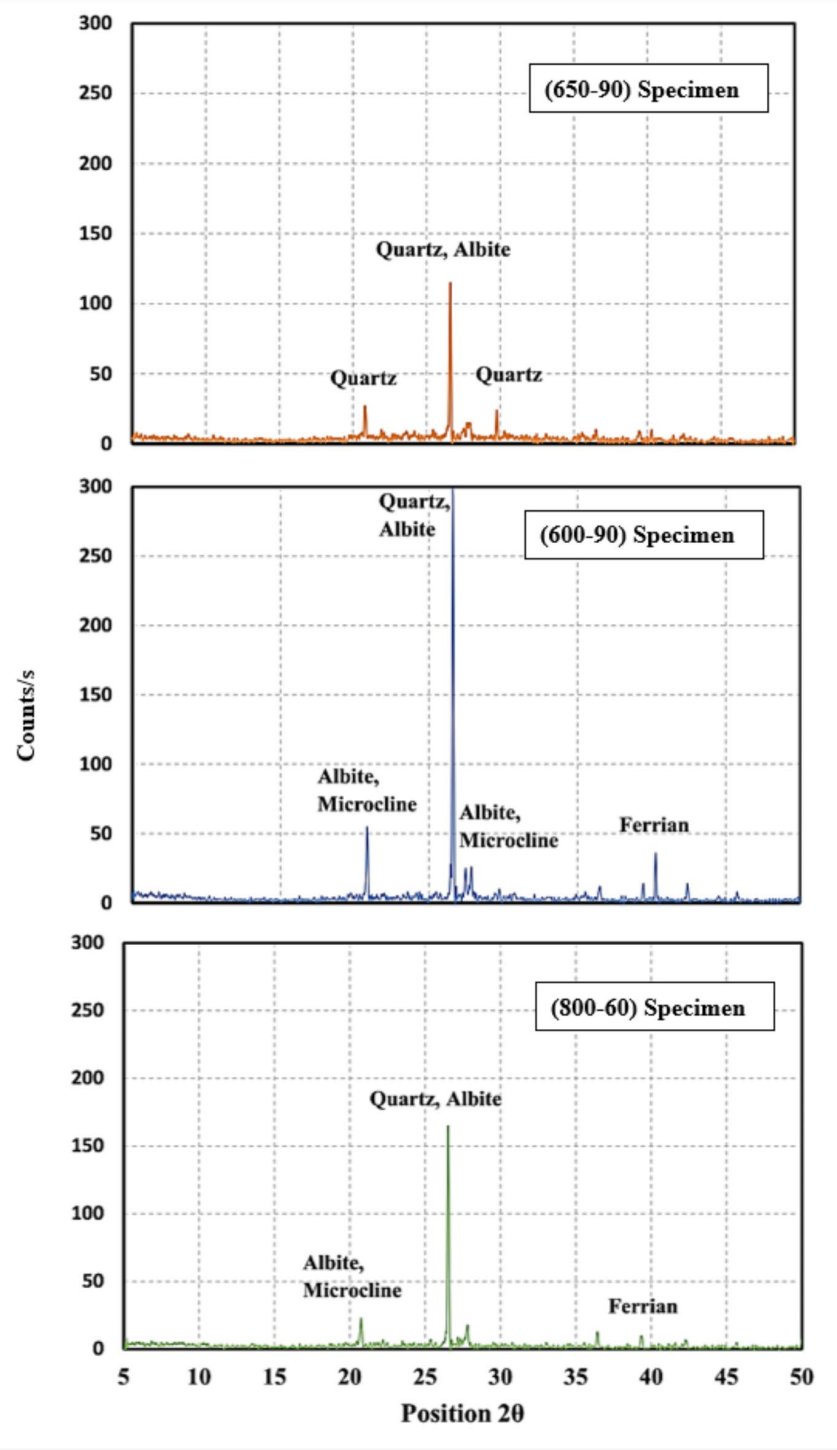
XRD test results.

**Fig. 8:**
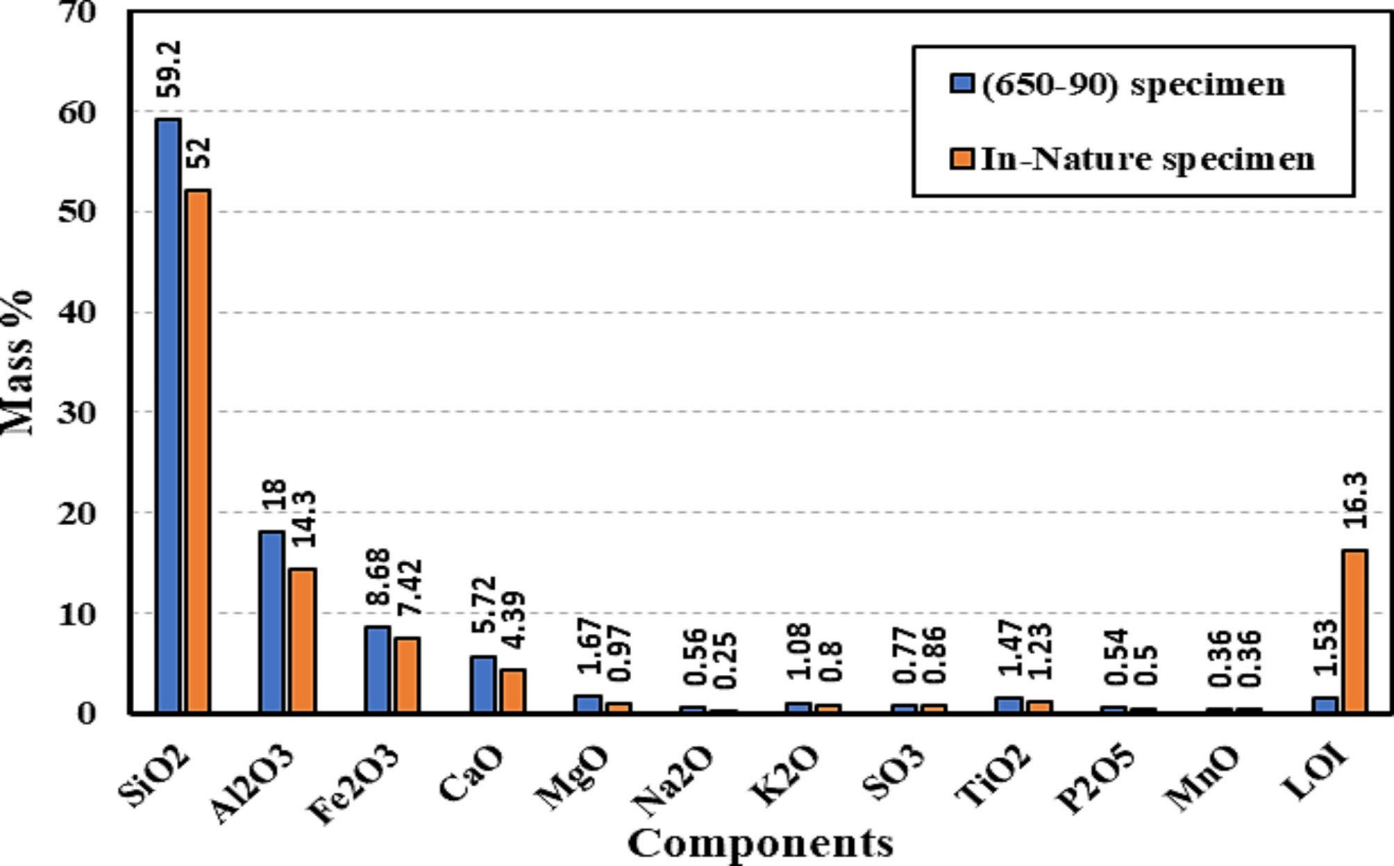
Comparison between the in-nature and (650 − 90) WTPS specimens in the XRF results.

The XRD test was conducted on three specimens based on the obtained PAS values; specimen (650−90) had the highest obtained PAS value, specimen (600−90) had the mean obtained PAS value, and specimen (800−60) had the lowest obtained PAS value. Calcination of the prepared WTPS powder at 650 °C for 90 min resulted in the disappearance of the peaks attributed to the presence of microcline (K Al Si_3_ O_8_) and the transformation to an amorphous material with fewer peaks attributed mainly to the presence of quartz at lower counts. The highest peak recorded for the (650−90) specimen was at 2θ equal to 26.6, which was attributed to quartz with a total of 110. The calcination of microcline produces high reactive surface area for the reaction with alkaline solutions or other binders and this leads to strong and durable geopolymer concrete. Microcline transforms when calcined from crystal structure to an amorphous structure with new compounds, illite. Illite is a layered silicate structure that is more suitable for chemical reactivity, with its enhanced surface area and microstructure allow for better bonding making it ideal for binder and construction applications^35–37^. Additionally, the XRD results showed the presence of albite (Na_0.98_ Ca_0.02;_ Al_2_ Si_2_O_5_ O_8_) but with ill-crystalline peaks, confirming that the calcined WTPS had an amorphous structure, as shown in Fig. 7. The formation of new compounds such as iron oxide (Fe_2_O_3_) and illite (2K_2_O 3MgO Al_2_O_3_ 24 Si O_2_ 12 H_2_O) resulted from the calcination of the WTPS at 650 °C. Upon comparing the XRD pattern of the (600−90) specimen shown in Fig. 7 to the pattern of the (650−90) specimen shown in Fig. 7, it can be observed that the specimen (600−90) has higher counts attributed to quartz, as shown in Fig. 7, than does the (650−90) specimen. Additionally, the microcline (K Al Si_3_ O_8_) and giniite, ferrian (Fe_5_ (PO_4_)_4_ (OH)_3_ 2 H_2_O) peaks were found in the (600−90) specimen at 2θ values equal to 29.83, 39.49, 40.31 and 40.47, respectively, where they almost disappeared in the (650−90) specimen. Additionally, by comparing the areas under the graphs for both specimens (650−90) and (600−90), it can be observed that the area attributed to specimen (650−90) is larger than that attributed to the other specimens, resembling more amorphous materials and confirming that the highest PAS value was achieved for specimen (650−90)^17,34^. The XRD pattern of the (800−60) specimen, shown in Fig. 7, which is associated with the lowest PAS, has shown higher peak counts than the (650−90) specimen with almost the same compounds as the (600−90) specimen but with a higher peak count for quartz at 2θ 26.61. This means that due to increasing the calcination temperature led to material recrystallization^34^. Additionally, upon comparing the area under the graph for the (800−60) specimens with that of the (600−90) and (650−90) specimens, it can be observed that the smallest area is governed by the (800−60) specimen, confirming the results obtained from the Chapelle test. The XRD test had proved that treated WTPS at 650 for 90 min provides an amorphous material suitable for chemical reactivity. Furthermore, an XRF test was carried out on the specimen (650−90) to assess the changes in chemical composition after the calcination process. The XRF test results presented in Table 7 reveal an increase in SiO_2_ and Al_2_O_3_ compared to those in the in-nature specimen Table 2; in total, 77.2%, the in-nature specimen had an increase in SiO_2_ and Al_2_O_3_ content of 16.44%. This means that there was an increase in the ability of the WTPS to be activated as a binder when calcinated, which is consistent with the findings of both the Chapelle test and the XRD test. Additionally, the loss on ignition (LOI) decreased from 16.3 to 1.53% after calcination, a reduction of 90.60%, which ensures the loss of organic and volatile materials from the treated WTPS. The increase in the SiO_2_ and Al_2_O_3_ content and the decrease in the LOI allows for better geopolymerization as the SiO_2_ and Al_2_O_3_ phases are more accessible and the potential of forming N-A-S-H (sodium alumino-silicate hydrate) gel is enhanced and sequentially the hardening and strength development of the material. Also, the removal of the inorganic materials allows the rest of the inorganic portion to become more homogeneous and structurally stable. This ensures that the treated WTPS is suitable for reactivity and to work as a binder in geopolymer material^37,38^. Figure 8 shows a comparison between the XRF results for both the in-nature and (650−90) WTPS specimens.

**Table 7:** XRF results for the (650−90) specimen.

Components	SiO_2_	Al_2_O_3_	Fe_2_O_3_	CaO	MgO	Na_2_O	K_2_O	SO_3_	TiO_2_	*P*_2_O_5_	MnO	LOI
Mass %	59.20	18.00	8.68	5.72	1.67	0.56	1.08	0.77	1.47	0.54	0.36	1.53

**Fig. 9:**
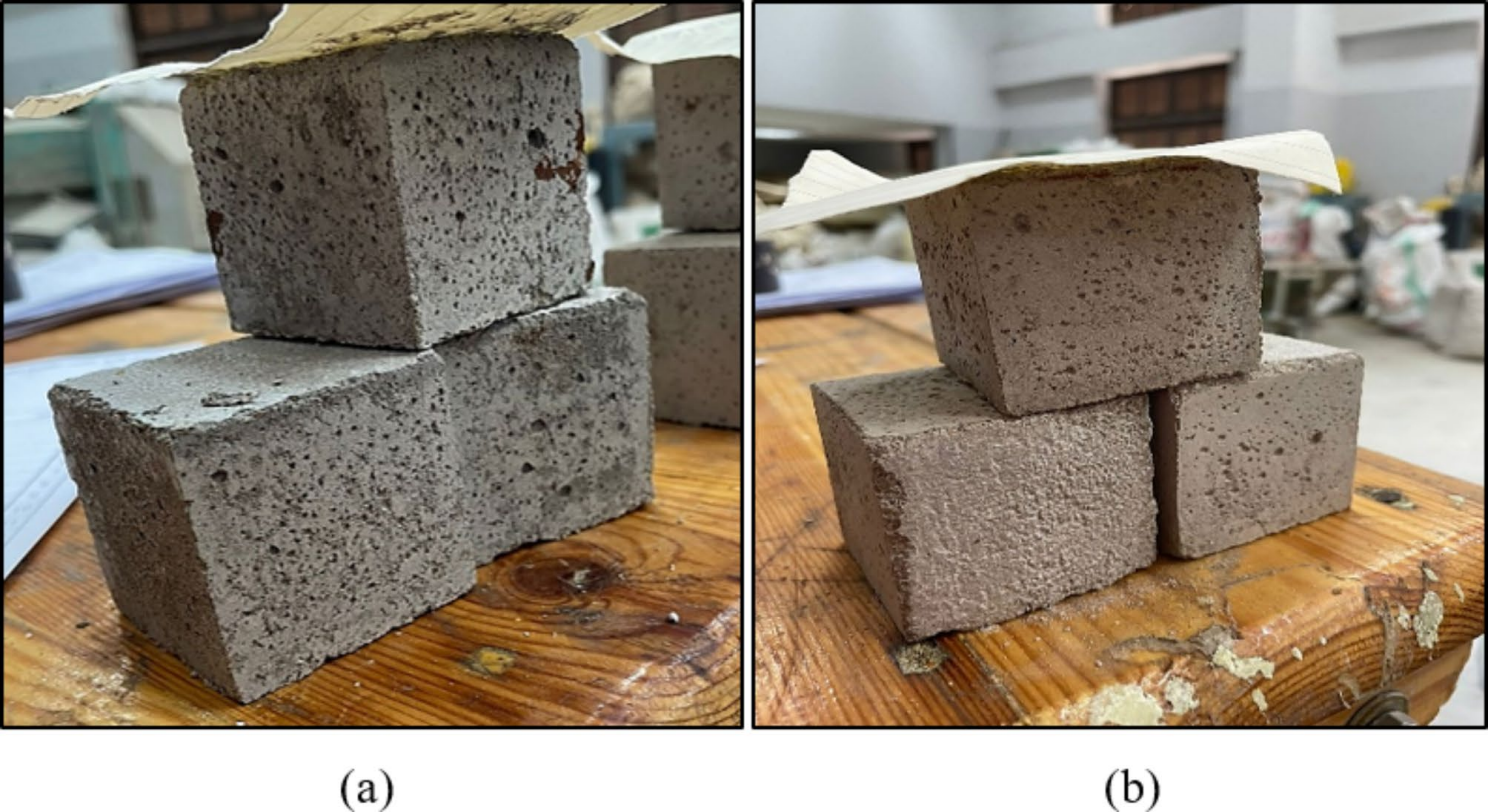
Shape of the performed cubic specimens. (**a**) Control mix cubes. (**b**) Test mix (650 − 90) cubes.

A strength activity index test was conducted on the (650−90) specimen, which had the highest PAS value (1194), to investigate its activation ability from the perspective of mechanical properties. The obtained strength activity index was approximately 145%, which indicates pozzolanic reactivity according to ASTM C311^26^. The recorded 7 day compressive strengths, used to calculate the strength activity index, were 3.72 MPa and 5.4 MPa for the control mix and the test mix (650−90) specimens, respectively. Figure 9 shows the shapes of the cubic specimens of both the control and test mixes. The recorded results of the strength activity index confirmed the findings of the Chapelle, XRD and XRF tests. The treated WTPS is proved to be activated and can work as a precursor in geopolymer material.

**Fig. 10:**
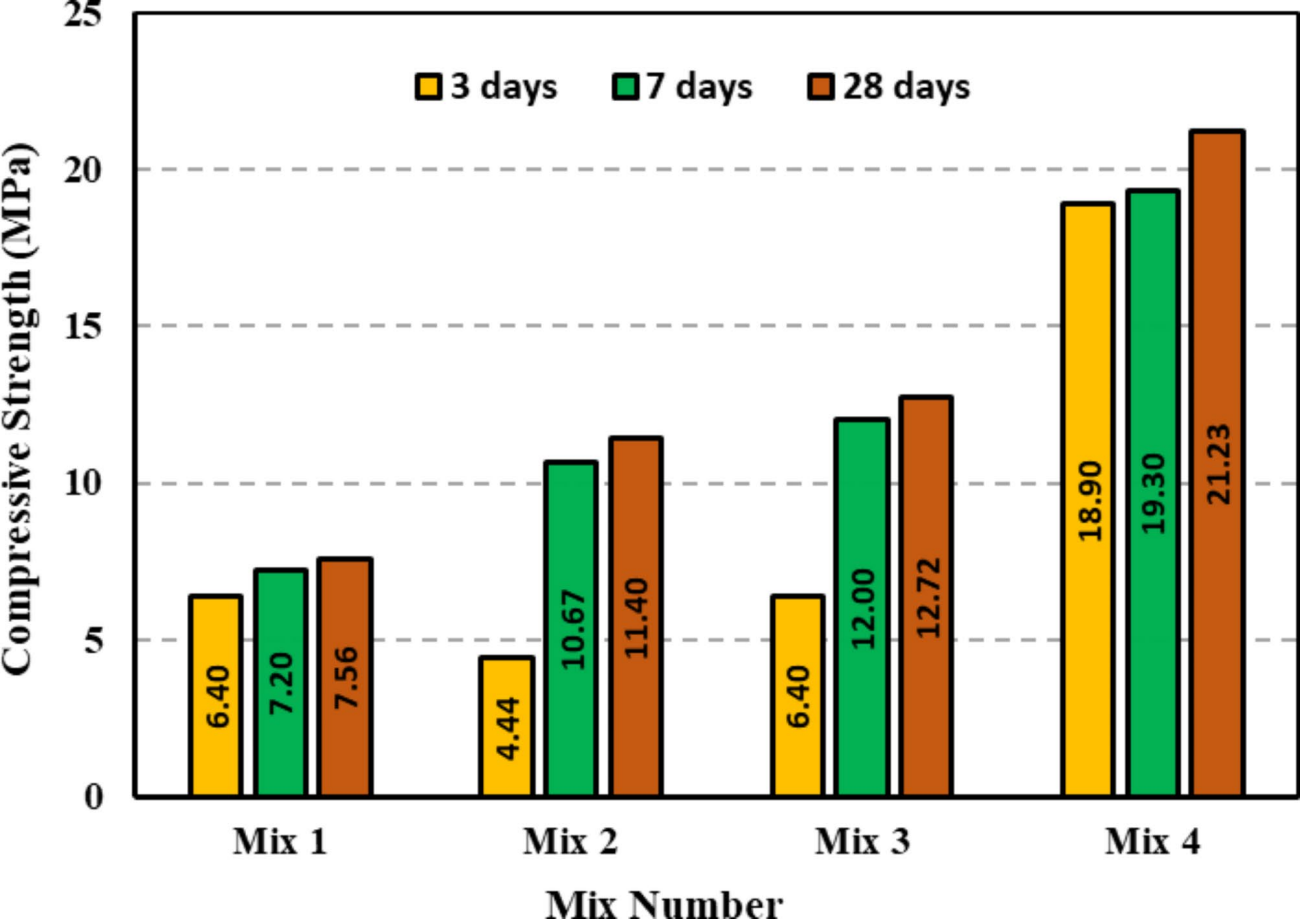
Compressive strength results of the four tested alkali-activated mixes.

The compressive strength test results for the four previously indicated mixtures at 3 days, 7 days and 28 days are presented in Fig. 10. By comparing the Mix 1 and Mix 2 results, it can be observed that increasing the Ms temperature did not enhance the compressive strength, and this can be attributed to the fact that a higher Ms causes diluting of the alkalinity of the medium with sodium silicate and consequently leads to less dissolution of the precursor during geopolymerization^39^. Additionally, heat curing was proven to be effective at increasing the compressive strength when used for the third mix, confirming the findings of previous studies, and this is due to the elimination of water in the fresh state, which results in a denser structure^28^. By mixing GGBFS with the WTPS powder, the compressive strength was significantly enhanced. When comparing Mix 2 to Mix 4, the compressive strength increased by 325%, 80% and 86% for the 3-day, 7-day and 28-day compressive strengths, respectively. This is due to the presence of CaO as a precursor with aluminosilicate, which results in the formation of C-A-S-H and C-S-H gels^5,32^. The first three mixes showed an increase in compressive strength between the 3 day and 7 day results, while for the fourth mix, there was almost no difference between the 3 day and 7 day compressive strengths. This is also because of the CaO found in GGBFS, which reacts quickly at the beginning of the process, forming the C-S-H gel^32^. The compressive strength results of the four mixes confirmed the activation of the WTPS powder and its ability to work as a binder.

## Energy and economic feasibility study

In this section the production process of WTPS is compared to the production process of cement in terms of cost and energy. The impact on the environment of geopolymers and OPC is evaluated by using life-cycle assessment analysis (LCA). This is the method that is used by industry and academics. LCA considers all of the product environmental impact from the acquisition of raw material till the final disposal. Previous studies have assessed the net CO_2_ savings and the Global Warming Potential (GWP) of geopolymers and compared it to OPC^1,40^. It is reported that geopolymers record a reduction in the net GWP by 96% in comparison with OPC^1^. Also, according to Australian Feedstocks, alkali activated concrete is recorded to have a reduction in CO_2_ emissions between 44 and 64%^29^. As mentioned in Sect. 2.2, the WTPS preparation process includes dewatering, grinding and firing processes. The energy consumed for each process is 0.0756^41^, 0.108^42^, 0.1296^43^ GJ/ton respectively with a total energy of 0.3132 GJ/ton. On the other hand, the total energy needed for cement production is approximately 3.2–6.3 GJ per ton^39^. The energy consumption of each process of WTPS production is derived from datasheets cited. The estimated cost of producing one ton of WTPS and cement were 1700^44^ and 3000^45^ EGP/ton respectively. The cost consumption of WTPS and cement were collected from local producers and sellers located in Egypt. The cost of the WTPS production processes is derived from the ASCE company, Egypt^44^ and the cost of one ton of cement is determined from Cemex, Egypt^45^.

Upon comparing the production energy consumption for WTPS and cement, the production of one ton of WTPS requires almost 92% less than the energy needed to produce one ton of cement. The reduction in energy consumption related to the WTPS reflects the decrease of the CO_2_ emissions during production. And this confirms with the sustainable development goals (SDGs)^7^. In terms of cost, WTPS production costs almost 50% less than the production cost of cement. It can be concluded that the WTPS provides an economic and energy efficient alternative to cement to be used in the construction era.

## Conclusion

This study aimed to determine the optimum treatment for water treatment plant sludge (WTPS) by calcination to develop WTPS as a precursor for alkali-activated materials. Additionally, the paper focused on carrying an economic and energy feasibility analysis for WTPS. Based on the analysis and discussion of the results obtained, the following conclusions can be drawn:

- WTPS can be developed as a precursor for alkali-activated materials by applying proper calcination treatment.
- Based on the Chapelle test (PAS value 1194), XRD test, XRF test and strength activity index test results (145%), calcination of the WTPS powder increased its pozzolanic reactivity, amorphous phases, aluminosilicate oxides and strength activity.
- The optimum calcination treatment of the WTPS powder, which achieved the best chemical and mechanical characteristics, was obtained by applying calcination regium at a maximum temperature of 650 °C for a 90 min duration.
- Based on the compressive strength, the calcined WTPS powder can be used to produce alkali-activated concrete with a desirable compressive strength around 21 MPa.
- WTPS provides an economic and energy-efficient alternative to cement as the production of WTPS requires 92% less energy and costs almost 50% less than the cement production.

## Data Availability

All data generated or analyzed during this study are included in this published article.
